# Understanding the successes and challenges of a social prescribing program for children and youth in Canada: a qualitative evaluation

**DOI:** 10.3389/fpubh.2026.1747222

**Published:** 2026-03-26

**Authors:** Caitlin Muhl, Isobel Fishman, Valeria Dimitrova, Ellen Song, Ashley Buffone, Samantha Kempe, Nicole Racine, Susan Bennett

**Affiliations:** 1 Vanier Social Pediatric Hub, Ottawa, ON, Canada; 2 Faculty of Health Sciences, Queen's University, Kingston, ON, Canada; 3 Faculty of Medicine, University of Ottawa, Ottawa, ON, Canada; 4 Faculty of Social Sciences, University of Ottawa, Ottawa, ON, Canada; 5 Children's Hospital of Eastern Ontario Research Institute, Ottawa, ON, Canada; 6 Children's Hospital of Eastern Ontario, Ottawa, ON, Canada

**Keywords:** challenges, children and youth, social prescribing, successes, upstream care

## Abstract

**Background:**

Social prescribing offers a tool to move care upstream to address the non-medical factors that impact health and wellbeing. In 2023, we launched a social prescribing program in a comprehensive, pediatric integrated health and social service hub in an underserved inner city neighborhood in Ottawa, Ontario, Canada. The program targeted children and youth with poor social health, including social isolation and loneliness. Program participants were paired with a connector, who worked with them to explore their individual needs, strengths, interests, and dreams. Together, they created a social prescription for a child and youth-friendly community activity. The connector then provided a supported referral to aid the program participant in successfully completing their social prescription. The program was piloted at the hub over a period of 10 months, during which time a program evaluation took place. As part of our program evaluation, we sought to explore the successes and challenges from the perspective of key stakeholder groups. Thus, the aim of this evaluation was to understand the successes and challenges of our child and youth social prescribing program from the perspective of program participants, caregivers, and staff members.

**Methods:**

A qualitative descriptive design was employed. Semi-structured interviews were conducted with 33 program participants, 30 caregivers, and five staff members. Interview questions centered around interviewees' experiences of the program. Interviews were audio recorded, transcribed verbatim, and anonymized. Data were thematically analyzed.

**Results:**

Successes included participant and caregiver satisfaction, enhancing the social connectedness of participants and families, addressing barriers to engagement, provider satisfaction, and community cohesion. Challenges included finding the right social prescription, addressing barriers to engagement, and managing operational constraints.

**Conclusion:**

This evaluation investigated the successes and challenges of our child and youth social prescribing program from the perspective of program participants, caregivers, and staff members. The findings of this evaluation add to the growing evidence base on social prescribing in pediatric populations in Canada.

## 1 Introduction

It was in 1975 that medical sociologist Irving Zola first told the story of a physician who was grappling with the challenges of modern medicine (1). The story goes as follows:

“*You know,” he said, “sometimes it feels like this. There I am standing by the shore of a swiftly flowing river and I hear the cry of a drowning man. So I jump into the river, put my arms around him, pull him to shore, and apply artificial respiration. Just when he begins to breathe, there is another cry for help. So I jump into the river, reach him, pull him to shore, apply artificial respiration, and then just as he begins to breathe, another cry for help. So back in the river again, reaching, pulling, applying, breathing, and then another yell. Again and again, without end, goes the sequence. You know, I am so busy jumping in, pulling them to shore, applying artificial respiration, that I have no time to see who the hell is upstream pushing them all in.”* ((1), p. 1)

This story, known as the stream metaphor, is one of the most popular and powerful concepts in public health (1). What this highlights is that health systems offer downstream solutions—medical interventions that treat problems rather than preventing them from happening in the first place. Even 50 years later, this remains the reality of health systems worldwide. As health system leaders come to terms with the inadequacies of this reactive approach to care, many are looking to social prescribing as a tool to move care upstream (2–4). Social prescribing is “a means for trusted individuals in clinical and community settings to identify that a person has non-medical, health-related social needs and to subsequently connect them to non-clinical supports and services within the community by co-producing a social prescription—a non-medical prescription, to improve health and wellbeing and to strengthen community connections” ((5), p. 9). Social prescribing signals a paradigm shift toward upstream care that addresses the non-medical factors that shape the vast majority of health and wellbeing (2–4, 6).

There is a growing body of evidence around the effectiveness of social prescribing in different settings and populations (2–4). Research suggests that this complex intervention may not only improve health and wellbeing but also reduce healthcare demand and cost (7–22). Much of the evidence that has been gathered to date pertains to adult social prescribing, with comparatively little evidence around child and youth social prescribing (23–27). Heeding calls to address this gap (25–28), several evaluations of child and youth social prescribing have emerged in recent years (29–42). These investigations have shed light on some of the successes and challenges of child and youth social prescribing, which are key insights for building a robust evidence base. As part of our program evaluation of our child and youth social prescribing program, we set out to explore the successes and challenges according to key stakeholder groups. Thus, the aim of this evaluation was to understand the successes and challenges of our child and youth social prescribing program from the perspective of program participants, caregivers, and staff members.

## 2 Methods

### 2.1 Design

A qualitative descriptive design was employed (43). Semi-structured interviews were conducted with key stakeholder groups to gain insights into their experiences of the program.

### 2.2 Setting

The social prescribing program took place in a comprehensive, pediatric integrated health and social service hub that was situated within a community services center based in Ottawa, Ontario, Canada. The former Vanier Social Pediatric Hub served children and youth who were 0–17 years of age, experiencing complex psychosocial issues, and either living or going to school in the Vanier neighborhood, which is one of the most socioeconomically deprived communities in Ottawa (44). This richly diverse urban neighborhood falls in the quintile of highest socioeconomic disadvantage on the socioeconomic index, with a high prevalence of unemployment, low income, housing insecurity, low educational attainment, and crime, as well as a high proportion of Francophone minoritized groups, immigrants and refugees, and ethnic and racial minority groups among its 18,000 residents. The hub embraced the community social pediatrics model that was developed by Montréal pediatrician, Dr. Gilles Julien (45). Social pediatrics is “a global, holistic, and multidisciplinary approach to child health—it considers the health of the child within the context of their society, environment, school, and family, integrating the physical, mental, and social dimensions of child health and development as well as care, prevention, and promotion of health and quality of life” (46).

### 2.3 Program

Under the community social pediatrics model, the hub team was already providing a holistic model of care. However, due to the socioeconomic challenges faced by the families presenting to the hub, the hub team found that they were spending most of their time addressing urgent basic needs of the caregivers like housing instability and food insecurity. The hub team saw the need to augment this crucial work with an offering that was focused on the personal dreams and wishes of the children and youth themselves. This led to the launch of a social prescribing program that was piloted at the hub over a period of 10 months (May 2023–February 2024) with the aim of promoting the health and wellbeing of the children and youth being served. The program was delivered in both official languages of Canada (English and French). There was a strong health equity focus to the program, with two key features being that the program targeted populations experiencing inequities and mitigated financial barriers such that families did not have to pay out of pocket for the social prescriptions.

Entry into the social prescribing pathway began at regular hub appointments, during which children and youth presented to the hub with their caregivers for assessment of their medical and social needs with the hub team. As part of this assessment, the hub team evaluated social health. This was done through conversations with the family rather than with a standardized screening tool. Those who were identified as having poor social health were eligible to participate in the social prescribing program, where social health is defined as “adequate quantity and quality of relationships in a particular context to meet an individual's need for meaningful human connection” ((47), p. 619). Once the hub team obtained consent from the caregiver and assent (ages 4–11)/consent (ages 12–17) from their child to participate in both the social prescribing program and the program evaluation, a referral was made to the program. The following reasons for referral were accepted for entry into the program: (1) feeling socially isolated; (2) feeling lonely; (3) lack of social skills; (4) lack of involvement in social activities; (5) lack of participation in opportunities in the community; (6) experiencing financial and/or transportation barriers to community engagement; and (7) poor sense of community belonging. Upon being referred to the program, the child was assigned to a connector. The program had three connectors, all of whom were hub staff. The child-connector dyad met within 1–2 weeks and then every few weeks thereafter, either at the hub, over the phone, in the home, or in the community—wherever the family preferred. Each session was up to 1 h in length. As a key part of the sessions, the connector worked with the child to explore their individual needs, strengths, interests, and dreams. By using the information that was gathered through this process to inform decision-making, the child-connector dyad co-produced a social prescription for a child and youth-friendly community activity. There were six different types of social prescriptions: (1) arts and culture; (2) physical activity; (3) time in nature; (4) career exploration; (5) practical skills; and (6) entertainment and leisure. Social prescriptions were written on a social prescription pad and, after being signed by both the child and connector, were given to the child in the same way that a medical prescription would be. The connector provided a supported referral and met with the child on an ongoing basis to offer encouragement and motivation, build rapport, monitor progress, and co-produce additional social prescriptions as needed. Through a feedback loop, the connector reported back to the rest of the hub team. Everything was documented in the electronic medical record, including referrals, sessions, and social prescriptions. The program is described in greater detail elsewhere.^1^

### 2.4 Participants

This evaluation consisted of a convenience sample of program participants (*n* = 33), caregivers (*n* = 30), and staff members (*n* = 5) involved in the social prescribing program. All program participants, caregivers, and staff members were eligible to participate.

### 2.5 Recruitment

Hub staff contacted families by phone to inform them about the opportunity to participate and to schedule an interview date and time for those who wished to participate. As for the recruitment of hub staff, they were contacted by a medical trainee via email to discuss the opportunity to participate and to schedule an interview date and time for those who wished to participate.

### 2.6 Data collection

At the time of enrollment in the program, caregivers completed an intake form on behalf of their child. Sociodemographic information collected through the form included age, gender identity, mother tongue, official language preference, country of birth, newcomer status, ethnicity/race, disability status, annual household income, and household composition.

Between November 2023 and February 2024, semi-structured interviews were conducted with program participants and caregivers at the 6-month mark of enrollment in the program. To mitigate the risk of interviewer bias and response bias, the interviews were conducted by medical trainees (IF, VD, ES, AB) who were not involved in the clinical care of the program participants nor the program development or implementation, except for the work they did behind the scenes to assist the connectors in arranging the social prescriptions. The medical trainees consisted of third- and fourth-year medical students (IF, VD, ES) and a pediatric resident (AB). All had prior experience conducting qualitative interviews. Given the wide age range and developmental diversity of program participants, two interview guides were developed—one for 4 to 7-year-olds (Supplementary material 1) and one for 8 to 17-year-olds (Supplementary material 2), with discretion given to the interviewers to select the most appropriate interview guide based on age and developmental ability. The interview guide for 4 to 7-year-olds had a corresponding interview activity (Supplementary material 3). The interview activity was a drawing exercise, whereby the child was asked to draw pictures of their connector and any social prescriptions they participated in. The drawings were used as an elicitation tool, as drawing is known to support memory performance and facilitate communication in interviews with children (48), particularly young children (49). A separate interview guide was developed for caregivers (Supplementary material 4).

In February 2024, semi-structured interviews were conducted with staff members. As with the family interviews, the staff interviews were conducted by a medical trainee (IF). An interview guide for staff members was developed (Supplementary material 5).

Interview questions centered around interviewees' experiences of the program. Interviews were conducted in either English or French and occurred either in person at the hub or over the phone. Family members were interviewed one at a time, while remaining together in the same room or on the same phone call. Staff members were interviewed individually. Program participant and caregiver interviews took approximately 20 min, while staff member interviews took approximately 30 min. Interviews were audio recorded, transcribed verbatim, and anonymized.

### 2.7 Data analysis

Sociodemographic data were analyzed using Microsoft Excel. Following the approach set out by Braun and Clarke (50), qualitative data were thematically analyzed, with data management supported by NVivo (Version 14; Lumivero, Burlington, MA, USA). This involved taking the following six steps: (1) familiarizing oneself with the data; (2) generating codes; (3) searching for themes; (4) reviewing themes; (5) defining and naming themes; and (6) producing a report (50). An inductive thematic analysis approach was employed, which is characterized by Braun and Clarke (50) as a bottom-up, data-driven approach that involves generating codes from the data itself rather than using a pre-existing coding frame. The lead investigator (CM) analyzed the data. The themes were presented to the senior investigator (SB) for review. Disagreements were resolved through discussion.

### 2.8 Reflexivity

Our team was mindful of our backgrounds, beliefs, and biases and how these might influence the evaluation process. In particular, we considered how our overlapping roles and responsibilities across program development, implementation, and evaluation might shape both our approach to data collection and data analysis. We critically reflected on these influences throughout the evaluation process, both individually and as a group, to ensure the integrity of our findings.

### 2.9 Ethical considerations

Since this work was conducted as part of a program evaluation, it was deemed to be a non-research activity and was therefore exempt from ethics approval by the Children's Hospital of Eastern Ontario (CHEO) Research Institute. Informed consent was obtained from the families (written and verbal consent) and staff members (verbal consent) involved in this evaluation. Families received a $25 CAD gift card as a token of appreciation for their time.

## 3 Results

Semi-structured interviews were conducted with 30 families, including 33 program participants and 30 caregivers. There were 33 families and 43 program participants in the pilot, meaning the recruitment rate for this evaluation was 91 and 77%, respectively. There were no notable differences between those who participated in the evaluation and those who did not. Interviews were also conducted with five staff members, all of whom were intimately involved in the delivery of the social prescribing program. As outlined in Table 1, 67% of program participants were 8–12 years of age (mean = 10 years of age), 55% were male, 33% had a mother tongue that was not one of the official languages of Canada (English and French), 30% were Francophone minoritized groups, 39% were born outside of North America, 30% were newcomers (immigrant or refugee in Canada <5 years), 85% were ethnic and racial minority groups, and 73% had a disability (e.g., attention deficit hyperactivity disorder, autism spectrum disorder). As outlined in Table 2, program participants came from families facing multiple socioeconomic challenges, with 60% of families reporting an annual household income of $39,999 CAD or less and 53% classifying themselves as a single caregiver household.

**Table 1:** Program participant characteristics (*N* = 33).

**Characteristic**	***N***	**%**
**Age**		
4–7	3	9.1
8–12	22	66.7
13–17	8	24.2
**Gender identity**		
Male	18	54.5
Female	15	45.5
**Mother tongue**		
Arabic	4	12.1
English	13	39.4
French	9	27.3
Other	7	21.2
**Official language preference**		
English	21	63.6
French	10	30.3
Both	2	6.1
**Region of birth**		
Africa	4	12.1
North America	20	60.6
Middle East	9	27.3
**Newcomer status**		
Yes	10	30.3
No	23	69.7
**Ethnicity/race**		
Asian	4	12.1
Black	14	33.3
Middle Eastern	6	18.2
White	5	15.2
Other	4	12.1
**Disability status**		
Developmental disability	13	39.4
Learning disability	4	12.1
Mental disability	4	12.1
Physical disability	3	9.1
None	9	27.3

**Table 2:** Family characteristics (*N* = 30).

**Characteristic**	***N***	**%**
**Annual household income (CAD)**		
$0–19,999	10	33.3
$20,000–39,999	8	26.7
$40,000+	5	16.7
No response	7	23.3
**Household composition**		
Single caregiver	16	53.3
Multiple caregivers	14	46.7

### 3.1 Successes

#### 3.1.1 Participant and caregiver satisfaction

Program participants expressed their satisfaction with the social prescribing program.

Interviewer: “*What was your favourite part of the social prescribing program?*”

Participant #16 (age 11): “*I like everything. It was so fun.”*

Program participants had a positive experience with their connector. Participant #28 (age 12) noted, “*[My connector] really supported me and I really appreciate that.”* while Caregiver #20 (7-year-old child) shared, “*[His connector] was awesome. He was so communicative and positive and eager to help. Yeah, I thought it was great. And [my child] liked him. We have a lot of trouble getting out of the house sometimes, but if I would say, ‘We're going to see [your connector]', it was easier.”* Program participants also had a positive experience with many of their social prescriptions. Participant #4 (age 13) shared, “*I really liked [the theatre classes], honestly. It was very fun.”* while Caregiver #12 (8-year-old child) remarked, “*[My child] didn't want the art classes to end.”* Caregivers were also satisfied with the program. They valued core components of the social prescribing model, including the “what matters to you” conversation and the co-production of the social prescription. Caregiver #20 (7-year-old child) shared, “*I liked how individual [the social prescribing program] is. It's very customized. So yeah, I like how individual it is and how everybody took the time to listen to what [my child] needed, and our family because it is our family that they're ultimately helping too.”* Caregivers also expressed their gratitude for this opportunity. Caregiver #18 (8-year-old child) remarked, “*We feel so blessed that we become part of [the social prescribing program].”* Staff members also took note of how grateful families were. Staff Member #2 shared, “*There's one little child whose mother was just practically in tears saying, ‘I can't believe you have given this opportunity to my son'. I mean really, you know, just so grateful, cause the kid was beaming. He absolutely loved it.”*

#### 3.1.2 Enhancing the social connectedness of participants and families

Program participants spoke about the fact that the social prescribing program helped them to get out into their community to experience new things and make new friends. Participant #32 (age 14) shared, “*[The social prescribing program] helped me to get out of like the house and stuff. Helped me not be so afraid of like, new experiences like classes and stuff like that*.” Caregivers also spoke about this. For example, Caregiver #6 (4-year-old child) shared, “*I think [the social prescribing program] has put a lot of meaning to [my child's] life. Like I said, it's got him out of the house more. It made him some friends and new experiences and just be out in the world a little bit more.”* while Caregiver #23 (14-year-old child) noted, “*I think [my child] learned that even when it's hard to get out and go and even when it feels like maybe she wants to just stay at home, that going and like overcoming that anxious feeling can lead to a really positive experience.”* Caregivers also expressed that the program made their family feel more connected to their community. For example, Caregiver #8 (9- and 12-year-old children) remarked, “*Now [my children] see the community a little bit different, more positive, because they experienced new things, and they think this community has something good.”* while Caregiver #30 (8-year-old child) shared, “*I didn't even know some of these things were going on because we don't have access to them… and I think we feel more of a belonging to the community now than before when we are just on our own and don't have access to anything.”*

#### 3.1.3 Addressing barriers to engagement

Staff members and caregivers highlighted that the social prescribing program was successful in addressing a number of barriers to engagement. They emphasized the effort that was made to address financial barriers to engagement and how this affected children living in poverty. Staff Member #2 remarked, “*There were several social prescriptions that stuck out because they were so joyful and they were, um, so happy and you pause and think, ‘If we weren't paying for this or if they weren't subsidizing this, there's no way this kid would ever have had the exposure'.” One* way that financial barriers to engagement were addressed was by covering the cost of the social prescriptions, which enabled program participants to engage in activities that their family would not otherwise have been able to afford. Caregiver #23 (14-year-old child) shared, “*We wouldn't have been able to afford to do [the socially prescribed activities] without having them been covered.”* Financial barriers to engagement were also addressed by covering the cost of transportation, which enabled program participants to get to and from their social prescribing sessions and socially prescribed activities. Caregiver #20 (7-year-old child) shared, “*There was always transportation provided. So that's a huge help for us because we can't get places unless we take the bus.”* Another way that the program successfully addressed barriers to engagement was by providing a supported referral, where the connector assumed responsibility for arranging the social prescriptions so that, as much as possible, families were not impacted by potential structural barriers they may have faced due to their socioeconomic circumstances. The connector did this by seeking out the relevant community organization, registering the program participant for the socially prescribed activity when applicable, obtaining ticket(s) for the socially prescribed activity when applicable, and liaising between the community organization and the family. Caregivers noted how helpful this was. For example, Caregiver #3 (13-year-old child) shared, “*[The connector] facilitated, like she talked to the [community organization], had it all set up for us. That was helpful.”* while Caregiver #16 (10- and 11-year-old children) remarked, “*[The connector] tell me like this program we have and like [my children] can go there. And I said, ‘I'm happy to hear this, but I have the problem to how to apply', and I say, ‘Like many days I am like confusion what to do and how to apply', and he say, ‘Oh, don't worry, I do everything'. And he helped me. He did everything.”*

#### 3.1.4 Provider satisfaction

Staff members expressed that the social prescribing program brought them joy in work. Staff Member #2 remarked, “*I find this work really joyful. Like to have a child tell you about what they dream of. And some of the things that come out of their mouths are just so incredibly adorable and sometimes very moving. And it just made me, you know, love the work more.”* Staff members reported that the program gave them a sense of purpose and fulfillment. Staff Member #3 shared, “*Yeah [the social prescribing program] was very fulfilling, and it was a good reminder that this is why I am doing this, right? This is the reason why I'm waking up in the morning and coming to serve the families and kids, so it really was like a very fulfilling experience I would say.”* Since much of their day was devoted to emotionally demanding work that centered around meeting families' basic needs, staff members expressed that the joy they experienced from the social prescribing program equipped them to get through the difficult parts of their day.

“*A lot of what is done is hard and very sad. It's a lot more uplifting to speak with this little soul of a person that is developing about things that are joyful than it is to be speaking with their families about putting food on the table, right? And so it's a bit like inoculating us so that we can do the hard parts, because at least we get to do something joyful, and I think that's really important. Every person providing pediatric care to children living in poverty should have some component of their work that is really joyful and hopeful to avoid burnout. It can't all be sad.”*—Staff Member #2

#### 3.1.5 Community cohesion

Staff members described a ripple effect of the program, whereby the act of linking families to their community served to strengthen the community ties of the people within their network.

“*[The social prescribing program] is impacting the kids. But I also think it's teaching not only the kids and their families, but also the people in their network about more of the services that are offered… I just would say that because it's making such a positive impact on the families and the families are all from Vanier and know other families in Vanier that it's really just like expanding that way, that networking.”*—Staff Member #1

Staff members also noted that the program strengthened the linkages between community organizations. They described how the program helped to break down siloes and bring organizations together for the shared purpose of supporting the children in the community.

“*[Social prescribing] is all about community engagement. I know that that's in the literature as well, that there's impacts on the individual and the community, and I can really see how that works now. [The social prescribing program] has been an incredible pathway to bring siloed activities and agencies into the fold for the purposes of helping children to reach their full potential.”*—Staff Member #4

### 3.2 Challenges

#### 3.2.1 Finding the right social prescription

Caregivers and staff members noted that there were occasional instances where finding the right social prescription was a challenge. They spoke about situations where the program participant did not enjoy their social prescription. For example, Caregiver #10 (7-year-old child) shared, “*So when we got home, on our way upstairs, [my child] said, ‘Mom, please don't take me [to nature camp] again. I don't wanna go there again. So many bugs'.”* while Caregiver #26 (7-year-old child) remarked, “*[Dance class] wasn't really [my child's] thing… all he wanted to do the entire time is leave the room.”* Although this was acknowledged as being a challenge, it was also seen as being a valuable opportunity for self-discovery. Staff Member #1 explained, “*I think even though [the social prescription] didn't go well, it's still a super great learning experience to learn what you like versus what you don't like.”* Staff members spoke about their persistence and determination in working with program participants to try different social prescriptions until they found one they liked.

“*I loved, you know, [this child], who had a couple of social prescriptions that were not suitable, that he didn't like. And this was challenging, cause I thought, ‘Okay, well we're not gonna let this, we're gonna keep going'. And so when he identified that he really wanted to grow up and be a policeman and know a policeman, and when the [police station visit] finally happened, [the child] was over the moon.”*—Staff Member #2

Caregivers and staff members emphasized the fact that, much like how it can take time to find the right medical prescription, it can also take time to find the right social prescription. Caregiver #20 (7-year-old child), who had a particularly challenging time with this, remarked, “*In the same way that medical prescribing isn't always perfect the first time, social prescribing isn't always going to be perfect the first time either, like the point is we're trying.”*

#### 3.2.2 Addressing barriers to engagement

Staff members highlighted that they were not always successful in addressing barriers to engagement. One component of this was the barriers pertaining to the complex health issues of the program participants, which staff could not always address despite their best efforts.

“*So the example of the child with seizures, whose seizures were triggered by cold. So we went and got taxi vouchers so that mom could in fact bring the child. Now this is a non-verbal child with medical complications, for whom it would be really difficult to bring this child, in a wheelchair, to the National Arts Centre in downtown Ottawa in the winter, like it's complicated. So we tried to address those barriers, and we weren't successful, and I think that in itself is an important learning, because we could all, you know, do our best thinking to resolve barriers and be unsuccessful at that.”*—Staff Member #2

Another aspect of this was the barriers pertaining to caregiver engagement. Since program participants were entirely dependent on their caregivers, this in turn limited their engagement.

“*Sometimes we would have challenges from the parents to make it happen. Because at the end of the day, they are the adults, so if they're not allowing their kids to participate in an activity or if they can't take the kid to the activity or just make some time to go and pick them up, that makes it hard to actually have the social prescribing happen.”*—Staff Member #3

Poor caregiver engagement manifested in a number of ways, including difficulty getting in touch with the family, difficulty obtaining caregiver permission and required paperwork for their child to participate in their socially prescribed activity, and difficulty getting the caregiver to take their child to their social prescribing session or socially prescribed activity.

“*Something that's really hard is just getting the families to actually go. Obviously, you can't take it personally because there's other stuff going on, but it's really hard when you try your best and they, you know, they don't come and fill out a form that you need to write a social prescription even though you know the kid wants to do it or they do fill it out but then they just never go.”*—Staff Member #1

Staff members were quick to note that caregivers were well meaning and had good intentions but were limited by the barriers they faced. Staff Member #2 remarked, “*The answers to why the parents don't bring their children need to be nuanced. They love their children. They want what's best for their child. They may not be able to muster the energy, it may be too cold, it may be too hard to take a bus, or two buses.”* Caregivers spoke about the barriers they faced, which revolved around their socioeconomic circumstances and personal health issues. For example, Caregiver #24 (9-year-old child) shared, “*Right now, I'm a single parent; my husband's in jail, so I don't have the luxury of going swimming.”* while Caregiver #6 (4-year-old child) noted, “*[My child] was supposed to take [bike lessons] to learn to ride a bike, but unfortunately that didn't happen because I found out I was pregnant and I had severe morning sickness, so I couldn't take him to be outside in the heat to learn to ride a bike.”* Staff members also spoke about this. Staff Member #1 remarked, “*It's, you know, the families who can't come in for their appointment or can't make their appointment because mom is working or because dad has mental health struggles or because little brother got sick and is in the hospital now.”* while Staff Member #2 noted*, “There were cases in which the parents were struggling with their own mental health issues. Like one dad is taking, you know, medication to sleep and was not able to get up in the morning to actually show up and bring their kid to the activity.”* Despite their best efforts to address barriers to engagement, staff members shared that they had come to the realization that some of the barriers faced by families were simply beyond their control. Staff Member #1 remarked, “*I think some of the barriers that we have to understand in social prescribing is that there are so many things that come up in families lives that we (A) may not understand or (B) that are so out of our control that we can't do anything about it.”* while Staff Member #2 shared, “*I think acknowledging that even if you give something to somebody, even if you pave the way and try to address the barriers and try to be as thoughtful as you can to make it as easy as you can, that doesn't mean that you will be successful in implementing the social prescription.”*

#### 3.2.3 Managing operational constraints

Staff members spoke about the operational constraints, both internal and external, that their team had to navigate. Internally, the social prescribing team faced major human resource challenges. Staff members explained that their team was small and was operating at maximum capacity to deliver the program whilst juggling other responsibilities, and on top of this, there was staff turnover in two of the three connector positions. Staff Member #4 remarked, “*We're totally under-resourced.”* Staff members expressed feeling stretched thin as a result. They highlighted the resourcefulness of their team in navigating this challenge by drawing on clinical staff to fill the vacancies in the connector role and by leveraging the support of student volunteers. Externally, the social prescribing team faced challenges with community organizations. Staff members explained that they sometimes faced difficulty in corresponding with the community organizations to arrange the social prescriptions. Staff Member #3 shared, “*Some of them were not too responsive.”* Staff members also described situations where community organizations had application processes for subsidized programming that either had an unrealistic low-income threshold that did not reflect the current cost of living or had a requirement for families to provide sensitive financial information that was felt to be stigmatizing and burdensome. Staff members reported largely positive experiences with community organizations; however, when challenges did arise, they found this hindered the functioning of the social prescribing pathway.

## 4 Discussion

The aim of this evaluation was to understand the successes and challenges of our child and youth social prescribing program from the perspective of program participants, caregivers, and staff members. Thematic analysis revealed five “success” themes and three “challenge” themes.

One of the successes of the program was participant and caregiver satisfaction. Program participants had a positive experience with their connector and enjoyed many of their social prescriptions, while caregivers valued core components of the social prescribing model and felt grateful for this opportunity. These findings align with that of previous investigations of child and youth social prescribing, which have reported high levels of program participant and caregiver satisfaction (29–32). Another success of the program was enhancing the social connectedness of participants and families. The program enabled program participants, who were enrolled in the program due to having poor social health, to get out into their community to experience new things and make new friends. Previous studies on child and youth social prescribing have performed quantitative assessments of associated constructs, such as loneliness, and have also reported improvements (29, 33). The program also made families feel more connected to their community. This was also found in an evaluation of a family-based pediatric social prescribing program (37). These findings are important given the growing body of evidence surrounding the critical role that social connection plays in health and wellbeing and the fact that the highest rates of social isolation and loneliness are among young people (51). This was recently highlighted by the US Surgeon General, who declared that we are currently facing an epidemic of social isolation and loneliness (52). This was also positioned on the global health agenda by the World Health Organization (WHO) Commission on Social Connection, which recently released a landmark report that not only recognized social isolation and loneliness as being a global problem with serious impacts on morbidity and mortality but also pointed to social prescribing as a key solution (53). From a health equity standpoint, the program was successful in promoting health equity by reaching populations experiencing inequities and addressing a number of barriers to engagement, including financial and structural barriers. The ability of the program to address financial barriers by covering the cost of the social prescriptions and transportation is particularly significant as this has been a key challenge for other child and youth social prescribing programs (29, 30, 34, 37, 42). By taking a health equity approach, the program accessed populations experiencing inequities and then subsequently took action to mitigate the barriers they might otherwise face to participation. This is a key finding, as social prescribing is seen as being a health equity intervention (54); however, there is limited evidence to support this assertion. Particularly in Canada, where social prescribing is celebrated for being a pathway to health equity (55), this finding provides support for this claim. The ability of child and youth social prescribing to promote health equity has also been reported elsewhere (32). Another success of the program was provider satisfaction. The program brought joy in work to providers, giving them a sense of purpose and fulfillment and equipping them to get through the difficult parts of their day. Previous evaluations of child and youth social prescribing have also identified this as an outcome of the intervention (30–32, 34). This finding may be considered in relation to the issue of burnout in healthcare, as studies show that higher perceived clinic capacity to address social needs is associated with lower provider burnout (56–58). The program was also successful in promoting community cohesion. This was achieved via a ripple effect, whereby the act of linking families to their community served to strengthen the community ties of the people within their network. This was also achieved by strengthening the linkages between community organizations. This is a key finding, as social prescribing is thought to not only enhance individual wellbeing but also community wellbeing (3, 55); however, comparatively little evidence exists to support the latter. Particularly in Canada, where social prescribing is recognized for its ability to promote wellbeing at both the individual and community levels (55), this finding provides support for this claim. The ability of child and youth social prescribing to have positive impacts at the community level has also been reported elsewhere (32).

One of the challenges of the program was finding the right social prescription. Despite the fact that the social prescriptions were co-produced with program participants based on what matters to them, there were occasional instances where the program participant did not enjoy their social prescription. Through a process of trial and error, program participants tried different social prescriptions until they found one they liked. While this was acknowledged as being a challenge, it was also seen as being a valuable opportunity for self-discovery. Although the program was successful in addressing a number of barriers to engagement, this was also seen as being a challenge, as staff were not always able to do so despite their best efforts. This included barriers pertaining to the complex health issues of the program participants and the socioeconomic circumstances and personal health issues of the caregivers. Program participant and caregiver-related barriers to engagement have surfaced in previous investigations of child and youth social prescribing (29–31, 34, 42). In particular, caregiver engagement has been a key challenge. This issue may be examined through the lens of the COM-B model, which is a behavior change framework that outlines three necessary components for behavior to occur: (1) capability; (2) opportunity; and (3) motivation (59). In this case, even when a child was given the opportunity to engage in the social prescribing program and was motivated to do so, they were not capable of doing so without the support of their caregiver, which ultimately dictated their participation in the program. Another challenge of the program was managing operational constraints. Internally, the social prescribing team faced major human resource challenges due to being a small team that was operating at maximum capacity and dealing with staff turnover. Externally, the team faced challenges with community organizations in terms of having poor communication and problematic application processes. The issue of operational constraints has arisen in previous evaluations of child and youth social prescribing, where it has been noted that this impacts the quality of the service (29, 34, 42). This underscores the need for sufficient investment in social prescribing, both in the social prescribing program itself and in the community sector (27). Particularly in Canada, where the health system spends just 5% of its budget on prevention and health promotion (60) and the community sector is chronically underfunded (61), the operational constraints faced by programs such as ours are downstream effects of funding models that are not structured to support this type of work. This issue reflects broader structural health system constraints, including fragmented service delivery, workforce shortages, and reliance on short-term funding. Collectively, these system-level barriers limit the delivery, sustainability, and scaling of social prescribing. Public health policies that support adequate investment in upstream care and community are needed. This would not only create a supportive environment for social prescribing to thrive, but it would also be economically beneficial, as KPMG reports that every $1 CAD invested into social prescribing returns $4.43 CAD to society through improved wellbeing and reduced costs incurred on the health system and government (62).

While some of the successes and challenges of child and youth social prescribing have surfaced in previous studies, this has rarely been the primary analytic focus. This evaluation makes a meaningful contribution to the literature by intentionally centering the successes and challenges of our program as the sole focus of the analysis, allowing for in-depth exploration. Additionally, this evaluation employs innovative techniques, such as the use of drawing as an elicitation tool for younger children, which provides further evidence of its unique contribution to the literature.

### 4.1 Strengths and limitations

There are several strengths and limitations of this evaluation. One of the strengths is the triangulation of perspectives from multiple stakeholder groups, including program participants, caregivers, and staff members. Another strength is the number of participants, with 33 program participants, 30 caregivers, and five staff members, making this one of the largest qualitative evaluations of child and youth social prescribing to date. With respect to the families, there was significant diversity and representation from populations experiencing inequities. Taken together, the number and type of participants involved in this evaluation allows for a rich understanding of the phenomenon of interest. Additionally, the use of semi-structured interviews provided the right balance of structure and flexibility to gather meaningful insights on this topic.

With this being a program evaluation based in a community organization, this work was shaped by pragmatic needs and resource realities. There are associated limitations to note, including the overlapping roles and responsibilities of our team across program development, implementation, and evaluation, the limited follow-up duration and resultant focus on short-term successes and challenges of the program, the use of single coding for data analysis, and the lack of cultural analysis. These limitations reflect the real-world conditions in which this evaluation was conducted. Additionally, recognizing that this work consisted of a convenience sample from a single social prescribing program, the transferability of the findings to other contexts may be limited. Despite these limitations, the findings of this evaluation offer valuable insights about the successes and challenges of our child and youth social prescribing program.

### 4.2 Implications for research and practice

Our findings offer key insights for research and practice. With limited evidence on child and youth social prescribing (24), this evaluation adds to the evidence base by shedding light on the successes and challenges of this intervention. In future research, there is a need to explore this phenomenon further, particularly in terms of understanding what works, for whom, and in what circumstances. With respect to practice, the findings of this evaluation highlight various factors to consider in the design and delivery of other child and youth social prescribing programs. As we look to conduct a randomized controlled trial of social prescribing for children and youth on an outpatient mental health waitlist (63), our findings will inform our own research and practice.

## 5 Conclusion

In this evaluation, we examined the successes and challenges of our child and youth social prescribing program from the perspective of program participants, caregivers, and staff members. We identified a number of successes and challenges. The findings of this evaluation add to the growing evidence base around this topic.

## Data Availability

The raw data supporting the conclusions of this article will be made available by the authors, without undue reservation.

## Footnotes

1 Muhl C, Song E, Dimitrova V, Fishman I, Buffone A, Kempe S, et al. Leveraging the power of positive childhood experiences: a case study of a social prescribing program for children and youth in Canada. Manuscript submitted for publication.
