# Supplementary material for: Understanding the successes and challenges of a social prescribing program for children and youth in Canada: a qualitative evaluation
Source: Front Public Health. 2026 Mar 26;14:1747222. doi: 10.3389/fpubh.2026.1747222 (PMC13062229; doi:10.3389/fpubh.2026.1747222)
Supplement: Supplementary file 1 [file Table_1.docx]

**Supplementary Material 1**

**Interview Guide for Program Participants (Ages 4-7)**

**Introduction**

- Thank you for meeting with me. My name is [INSERT NAME], and I am a [INSERT ROLE] at the Vanier Social Pediatric Hub, where you have been participating in our social prescribing program.
- Today, I will be asking you about your experience with the program.
- We would like to make this program even better by asking you what you liked and what you did not like.
- Before we begin, I just want to remind you that you do not have to participate if you do not want to, and that you can stop participating at any time.
- There are no right or wrong answers to the questions. You do not have to answer questions that you do not want to answer.
- I will be recording this conversation so that the people who are trying to make this program even better can listen to it to learn about your experience with the program. Is that okay with you?
- We promise to keep your information safe.
- Do you have any questions for me before we begin?

**Interview Questions**

1. Do you remember [INSERT NAME OF CONNECTOR]? Can you draw a picture of your session with [INSERT NAME OF CONNECTOR]? Can you tell me about your picture? How did [INSERT NAME OF CONNECTOR] make you feel? Why did you feel [INSERT EMOTION]? What did you like about spending time with [INSERT NAME OF CONNECTOR]? What did you not like about spending time with [INSERT NAME OF CONNECTOR]?
2. Do you remember [INSERT NAME OF SOCIAL PRESCRIPTION]? Can you draw a picture of when you [INSERT NAME OF SOCIAL PRESCRIPTION]? Can you tell me about your picture? How did [INSERT NAME OF SOCIAL PRESCRIPTION] make you feel? Why did you feel [INSERT EMOTION]? What did you like about [INSERT NAME OF SOCIAL PRESCRIPTION]? What did you not like about [INSERT NAME OF SOCIAL PRESCRIPTION]?
3. Thinking about [INSERT NAME OF SOCIAL PRESCRIPTION], was there an adult at this activity who you liked spending time with?

- IF YES: Do you remember their name? What did [INSERT NAME OF PERSON] do with you? What did you like about spending time with [INSERT NAME OF PERSON]?
