# Supplementary material for: Understanding the successes and challenges of a social prescribing program for children and youth in Canada: a qualitative evaluation
Source: Front Public Health. 2026 Mar 26;14:1747222. doi: 10.3389/fpubh.2026.1747222 (PMC13062229; doi:10.3389/fpubh.2026.1747222)
Supplement: Supplementary file 2 [file Table_2.docx]

**Supplementary Material 2**

**Interview Guide for Program Participants (Ages 8-17)**

**Introduction**

- Thank you for meeting with me. My name is [INSERT NAME], and I am a [INSERT ROLE] at the Vanier Social Pediatric Hub, where you have been participating in our social prescribing program.
- Today, I will be asking you about your experience with the program.
- We would like to make this program even better by asking you what you liked and what you did not like.
- Before we begin, I just want to remind you that you do not have to participate if you do not want to, and that you can stop participating at any time.
- There are no right or wrong answers to the questions. You do not have to answer questions that you do not want to answer.
- I will be recording this conversation so that the people who are trying to make this program even better can listen to it to learn about your experience with the program. Is that okay with you?
- We promise to keep your information safe.
- Do you have any questions for me before we begin?

**Process Evaluation**

1. Can you tell me about the social prescribing program that you participated in?
2. What did you like about the social prescribing program?

- What was your favourite part of the social prescribing program? Why?

1. What did you not like about the social prescribing program?

- What was your least favourite part of the social prescribing program? Why?
- What suggestions do you have for improving the social prescribing program?

1. Can you tell me about your experience with [INSERT NAME OF CONNECTOR]?

- What did you enjoy about your experience with your connector? Why?
- What did you not enjoy about your experience with your connector? Why?
- Do you feel that your connector listened to you? Explain.
- Do you feel that your connector focused on what matters to you? Explain.
- Do you feel that your connector involved you in making decisions? Explain.
- Do you feel that your connector supported you to achieve your goals? Explain.

1. Can you tell me about your experience [INSERT NAME OF SOCIAL PRESCRIPTION]?

- What did you enjoy about this experience? Why?
- What did you not enjoy about this experience? Why?
- Do you feel that this experience was useful for you? Explain.
- Do you feel that this experience matched your interests? Explain.
- Thinking about [INSERT NAME OF SOCIAL PRESCRIPTION], was there an adult at this activity who you liked spending time with?
- IF YES: Do you remember their name? What did [INSERT NAME OF PERSON] do with you? What did you like about spending time with [INSERT NAME OF PERSON]?
- IF NO, move on to the next question.

**Outcome Evaluation**

1. Has your participation in the social prescribing program changed how you are feeling? Explain.
2. Have you gained or learned anything by participating in the social prescribing program? Explain. **Probing Questions:** Have you made new friends? Have you learned something new? Have you gained a new skill?
3. Do you feel that you have a greater sense of belonging to your community now that you have participated in the social prescribing program? Explain.
4. Has your participation in the social prescribing program changed how you feel about taking care of your health? Explain.
5. Has your participation in the social prescribing program impacted your life in any other way? Explain.

**Other**

1. Is there anything else you want to tell us about the social prescribing program?

**Probing Questions:**

- Can you tell me a little bit more about that?
- Can you give me an example of what you mean by…?
