# Supplementary material for: Understanding the successes and challenges of a social prescribing program for children and youth in Canada: a qualitative evaluation
Source: Front Public Health. 2026 Mar 26;14:1747222. doi: 10.3389/fpubh.2026.1747222 (PMC13062229; doi:10.3389/fpubh.2026.1747222)
Supplement: Supplementary file 3 [file Table_3.docx]

**Supplementary Material 3**

**Interview Activity for Program Participants (Ages 4-7)**

My name is ________________________________________

This is a picture of me and ____________________________


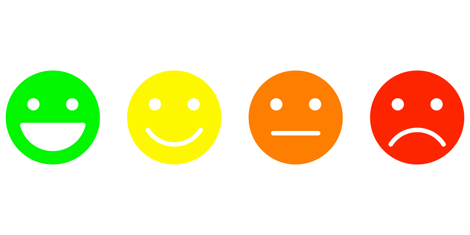


|  |
| --- |

____________________________ made me feel

This is a picture of ____________________________


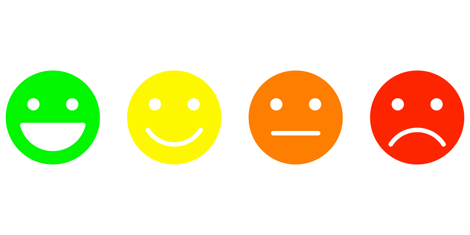


|  |
| --- |

____________________________ made me feel
