# Supplementary material for: Understanding the successes and challenges of a social prescribing program for children and youth in Canada: a qualitative evaluation
Source: Front Public Health. 2026 Mar 26;14:1747222. doi: 10.3389/fpubh.2026.1747222 (PMC13062229; doi:10.3389/fpubh.2026.1747222)
Supplement: Supplementary file 4 [file Table_4.docx]

**Supplementary Material 4**

**Interview Guide for Caregivers**

**Introduction**

- Thank you for meeting with me. My name is [INSERT NAME], and I am a [INSERT ROLE] at the Vanier Social Pediatric Hub, where your child has been participating in our social prescribing program.
- Today, I will be asking you about your child’s experience with the program.
- We would like to make this program even better by asking you what your child liked and did not like.
- Before we begin, I just want to remind you that you do not have to participate if you do not want to, and that you can stop participating at any time.
- There are no right or wrong answers to the questions. You do not have to answer questions that you do not want to answer.
- I will be recording this conversation so that the people who are trying to make this program even better can listen to it to learn about your child’s experience with the program. Is that okay with you?
- We promise to keep your information safe.
- Do you have any questions for me before we begin?

**Process Evaluation**

1. Can you tell me about the social prescribing program that your child participated in?
2. What did you like about the social prescribing program? What did your child like?

- What was your favourite part of the social prescribing program? Why? What about your child?

1. What did you not like about the social prescribing program? What did your child not like?

- What was your least favourite part of the social prescribing program? Why? What about your child?
- What suggestions do you have for improving the social prescribing program?

1. Can you tell me about your child’s experience with [INSERT NAME OF CONNECTOR]?

- What did your child enjoy about their experience with the connector? Why?
- What did your child not enjoy about their experience with the connector? Why?
- Do you feel that the connector listened to your child? Explain.
- Do you feel that the connector focused on what matters to your child? Explain.
- Do you feel that the connector involved your child in making decisions? Explain.
- Do you feel that the connector supported your child to achieve their goals? Explain.
- Do you feel that the connector did a good job of making sure that your child was able to participate in the social prescribing program? **Probing Questions:** Did they conduct home visits? Did they cover the cost of transportation? Did they cover the cost of the social prescription? Did they go with your child?

1. Can you tell me about your child’s experience [INSERT NAME OF SOCIAL PRESCRIPTION]?

- What did your child enjoy about this experience? Why?
- What did your child not enjoy about this experience? Why?
- Do you feel that this experience was useful for your child? Explain.
- Do you feel that this experience matched your child’s interests? Explain.
- Thinking about [INSERT NAME OF SOCIAL PRESCRIPTION], was there an adult at this activity who your child enjoyed spending time with?
- IF YES: Do you know their name? What did [INSERT NAME OF PERSON] do with your child? Did your child tell you what they enjoyed about spending time with [INSERT NAME OF PERSON]?
- IF NO, move on to the next question.

**Outcome Evaluation**

1. Has your child’s participation in the social prescribing program changed how they are feeling? Explain.
2. Has your child gained or learned anything by participating in the social prescribing program? Explain. **Probing Questions:** Have they made new friends? Have they learned something new? Have they gained a new skill?
3. Do you feel that your child has a greater sense of belonging to their community now that they have participated in the social prescribing program? Explain.
4. Has your child’s participation in the social prescribing program changed how they feel about taking care of their health? Explain.
5. Has your child’s participation in the social prescribing program impacted their life in any other way? Explain.

**Other**

1. Is there anything else you want to tell us about the social prescribing program?

**Probing Questions:**

- Can you tell me a little bit more about that?
- Can you give me an example of what you mean by…?
