# Supplementary material for: Understanding the successes and challenges of a social prescribing program for children and youth in Canada: a qualitative evaluation
Source: Front Public Health. 2026 Mar 26;14:1747222. doi: 10.3389/fpubh.2026.1747222 (PMC13062229; doi:10.3389/fpubh.2026.1747222)
Supplement: Supplementary file 5 [file Table_5.docx]

**Supplementary Material 5**

**Interview Guide for Staff Members**

| **Question** | **Probes** |
| --- | --- |
| 1. Can you tell me about your experience with the Vanier Social Pediatric Hub’s social prescribing program? |  |
| 1. What have you liked about the Hub’s social prescribing program? | - What has been your most favourite part of the social prescribing program? |
| 1. What have you not liked about the Hub’s social prescribing program? | - What has been your least favourite part of the social prescribing program? - What suggestions do you have for improving the social prescribing program? |
| 1. In what ways has the Hub’s social prescribing program impacted your role? | - How has this changed how you work? What are you doing differently? - Has the social prescribing program made it easier or harder to effectively do your job? How so? - Have you acquired any new knowledge or skills as a result of the program? |
| 1. In what ways has the social prescribing program impacted the Vanier Social Pediatric Hub? | - Do you think that the social prescribing program has enhanced the efforts of the Vanier Social Pediatric Hub to provide holistic care to clients? If yes, how so? If no, what makes you say that? |
| 1. In what ways has the Hub’s social prescribing program impacted the Vanier community? | - Do you think that the social prescribing program has been beneficial to the Vanier community? If yes, how so? If no, what makes you say that? |
| 1. Can you share your experience with the new tools and approaches that have been introduced for the social prescribing program? | - Have the tools and approaches been useful? - What has worked? What has not worked? - Has it been useful to standardize/formalize the process with these tools and approaches? |
| 1. Can you share your experience with co-producing social prescriptions with clients and focusing on what matters to them? |  |
| 1. What has been the impact of the community partnerships that have been established as a result of the social prescribing program? |  |
| 1. An important aspect of social prescribing is taking action to address any barriers that clients may face in completing their social prescriptions. Have you done this in the social prescribing program? If so, how? |  |
| 1. One of the benefits of social prescribing found in the literature has been a greater sense of purpose and enjoyment in work in service providers. Do you feel that this reflects your experience with the Hub’s social prescribing program? | - If not, can you explain why not? - If so, can you explain how? |
| 1. Please describe any memorable experiences you have had with children, youth or their caregivers who have gone through the Hub’s social prescribing program. | - What made this memorable? - Do you think such an experience would have happened without the social prescribing program? |
| 1. Can you share some of the challenges of the Hub’s social prescribing program? |  |
| 1. Can you share some of the successes of the Hub’s social prescribing program? |  |
| 1. Can you share some lessons learned from the Hub’s social prescribing program? |  |
| 1. Is there anything else you want to tell us about the social prescribing program? |  |
